# Supplementary material for: Personalized care planning and shared decision making in collaborative care programs for depression and anxiety disorders: A systematic review
Source: PLoS One. 2022 Jun 10;17(6):e0268649. doi: 10.1371/journal.pone.0268649 (PMC9187074; doi:10.1371/journal.pone.0268649)
Supplement: S1 File — (DOCX) [file pone.0268649.s001.docx]

**Search 1. Cochrane CCDAN references database**

#1 (Depress* or Dysthymi* or Anxiety or Anxious or panic or phobi* or PTSD or post-trauma or “post trauma” or panic or OCD or obsessi* or compulsi* or GAD):TI,AB,KY AND SR-DEPRESSN:CC

#2 (collaborat* or coordinat* or co-ordonat* or shared or integrat* or stepped or systematic) AND (care or healthcare or “health care” or working or intervention* or service or model or effort* or manage*) or Depress* or Dysthymi* or Anxiety or Anxious or panic or phobi* or PTSD or post-trauma or “post trauma” or panic or OCD or obsessi* or compulsi* or GAD AND SR-DEPRESSN:CC 1717

#3 (augment* or enhance*) AND (care* or healthcare or “health care” or communicat*) AND SR-DEPRESSN:CC

#4 “care manage*” or “case manage*” or “chronic care” or “complex intervention” or “cooperative behav*” or “co-operative behave*” or “joint working” or pathway or interprofessional or inter-professional or interdisciplinary or inter-disciplinary or multidiscipline* or multi-disciplin* or multiprofession* or multi-profession* or transdisciplin* or trans-disciplin* or multifacet* or multi-facet* or “multiple intervention*” or “multi-intervention*” or “organisational intervention*” or “organizational intervention*” or “interpersonal relation*” or “inter-institutional relation*” or “consultation liaison” or algorithm* or “treatment guideline*” or “treatment protocol*” or “treatment delivery” or “treatment model” or adherence or compliance or concordance or “patient care team” or “patient care management” or “patient care planning” or “case management” or “managed care program*” or “delivery of healthcare” or “continuity of patient care” or “professional-patient relations” or “interprofessional relations” AND SR-DEPRESSN:CC

#5 #2 OR #3 OR #4

#6 #1 AND #5

**Search 2. Cochrane CCDAN studies database**

#1 (Depress* or Dysthymi* or Anxiety or Anxious or panic or phobi* or obsessi* or compulsi* or post-traumatic):TI,AB,KY AND SR-DEPRESSN:CC

#2 (“care manag*” or “case manage*” or “collaborat* or “disease manag* or “enhanced care” or “managed care” or multicomponent or multi-component or multidisciplinary or multidisciplinary or stepped):TI,AB,KY AND SR-DEPRESSN:CC

#3 #1 AND #2

**Search 3. CINAHL database – Replication of 2012 Cochrane review**

| **#** | **Query** |
| --- | --- |
| S25 | S12 AND S23 AND S24 |
| S24 | MH “Quantitative Studies” OR MH ”Clinical Trials+“ OR MH ”Random Assignment“ OR PT ”Cinical Trial“ OR ( TI trial* or AB trial* ) OR ( TI ((singl* or doubl* or trebl* or tripl*) and (blind* or dummy or mask)) ) OR ( AB ((singl* or doubl* or trebl* or tripl*) and (blind* or dummy or mask)) ) OR ( TI (randomi?ed) or AB (randomi?ed) ) OR ( TI (random* and (allocat* or assign*)) or AB (random* and (allocat* or assign*)) ) OR ( TI (control* trial* or control* stud* or control group*) or AB (control* trial* or control* stud* or control group*) ) |
| S23 | S13 OR S14 OR S15 OR S16 OR S17 OR S18 OR S19 OR S20 OR S21 OR S22 |
| S22 | ( TI (care manage* or chronic care* or complex intervention* or cooperative behav* or co-operative behav* or joint working or pathway or interprofessional or inter-professional or interdisciplinary or inter-disciplinary or multidisciplin* or multi-disciplin* or multiprofession* or multi-profession* or transdisciplin* or trans-disciplin* or multifacet* or multi-facet* or complex intervention* or multiple intervention* or multi-intervention* or organisational intervention* or organizational intervention* or interpersonal relation” or inter-personal relation* or interinstitutional relation* or inter-institutional relation* or consultation liaison or algorithm* or treatment guideline* or treatment protocol* or treatment delivery or treatment model or adherence or compliance or concordance or patient care team or patient care management or patient care planning or case management or managed care program* or (healthcare N3 delivery) or (continuity N3 care) or professional-patient relations or interprofessional relations or inter-professional relations) ) OR ( AB (care manage* or chronic care* or complex intervention* or cooperative behav* or co-operative behav* or joint working or pathway or interprofessional or inter-professional or interdisciplinary or inter-disciplinary or multidisciplin* or multi-disciplin* or multiprofession* or multi-profession* or transdisciplin* or trans-disciplin* or multifacet* or multi-facet* or complex intervention* or multiple intervention* or multi-intervention* or organisational intervention* or organizational intervention* or interpersonal relation” or inter-personal relation* or interinstitutional relation* or inter-institutional relation* or consultation liaison or algorithm* or treatment guideline* or treatment protocol* or treatment delivery or treatment model or adherence or compliance or concordance or patient care team or patient care management or patient care planning or case management or managed care program* or (healthcare N3 delivery) or (continuity N3 care) or professional-patient relations or interprofessional relations or inter-professional relations) ) |
| S21 | TI "enhance* care*" or AB "enhance* care*" or TI "enhance* health*" or AB "enhance* health*" or TI "enhance* communicat*" or AB "enhance* communicat*" |
| S20 | TI "augment* care*" or AB "augment* care*" or TI "augment* health*" or AB "augment* health*" or TI "augment* communicat*" or AB "augment* communicat*" |
| S19 | TI "systematic care" or AB "systematic care" or TI "systematic health*" or AB "systematic health*" or TI "systematic work*" or AB "systematic work*" or TI "systematic interven*" or AB "systematic interven*" or TI "systematic service*" or AB "systematic service*" or TI "systematic model*" or AB "systematic model*" or TI "systematic effort*" or AB "systematic effort*" or TI "systematic manag*" or AB "systematic manag*" |
| S18 | TI "stepped care" or AB "stepped care" or TI "stepped health*" or AB "stepped health*" or TI "stepped work*" or AB "stepped work*" or TI "stepped interven*" or AB "stepped interven*" or TI "stepped service*" or AB "stepped service*" or TI "stepped model*" or AB "stepped model*" or TI "stepped effort*" or AB "stepped effort*" or TI "stepped manag*" or AB "stepped manag*" |
| S17 | TI "integrat* care" or AB "integrat* care" or TI "integrat* health*" or AB "integrat* health*" or TI "integrat* work*" or AB "integrat* work*" or TI "integrat* interven*" or AB "integrat* interven*" or TI "integrat* service*" or AB "integrat* service*" or TI "integrat* model*" or AB "integrat* model*" or TI "inte-grat* effort*" or AB "inte-grat* effort*" or TI "integrat* manag*" or AB "integrat* manag*" |
| S16 | TI "shared care" or AB "shared care" or TI "shared health*" or AB "shared health*" or TI "shared work*" or AB "shared work*" or TI "shared interven*" or AB "shared interven*" or TI "shared service*" or AB "shared service*" or TI "shared model*" or AB "shared model*" or TI "shared effort*" or AB "shared effort*" or TI "shared manag*" or AB "shared manag*" |
| S15 | TI "coordinat* care" or AB "coordinat* care" or TI "coordinat* health*" or AB "coordinat* health*" or TI "coordinat* work*" or AB "coordinat* work*" or TI "coordinat* interven*" or AB "coordinat* interven*" or TI "coordinat* service*" or AB "coordinat* service*" or TI "coordinat* model*" or AB "coordinat* model*" or TI "coordinat* effort*" or AB "coordinat* effort*" or TI "coordinat* manag*" or AB "coordinat* manag*" |
| S14 | TI "collaborat* care" AB "collaborat* care" or TI "collaborat* health*" or AB "collaborat* health*" or TI "collaborat* work*" or AB "collaborat* work* or TI "collaborat* interven*" or AB "collaborat* interven*" or TI "collaborat* service*" or AB "collaborat* service*" or TI "collaborat* model*" or AB "collaborat* model*" or TI "collaborat* effort*" or AB "collaborat* effort*" or TI "collaborat* manag*" or AB "collaborat* manag*" |
| S13 | MH “Case Mangement” OR MH Collaboration OR MH “Cooperative Behavior” OR MH “Interinstitutional Relations” OR MH “Interprofessional Relations” OR MH “Multidisciplinary Care Team” OR MH “Nurse-Physician Relations” OR MH ”Patient Compliance” OR MH “Patient Centered Care” OR MH “Pharmacists/UT” OR MH Teamwork |
| S12 | S1 OR S2 OR S3 OR S4 OR S5 OR S6 OR S7 OR S8 OR S9 OR S10 OR S11 |
| S11 | TI PTSD or AB PTSD or TI post-trauma* or AB post-trauma* or TI post trauma* or AB post-trauma* or TI postrauma* or AB postrauma* or TI posttrauma* or AB posttrauma* |
| S10 | TI GAD OR AB GAD |
| S9 | TI "social anxiety" or AB "social anxiety" |
| S8 | AB claustrophobi* OR TI claustrophobi* |
| S7 | AB agoraphobi* OR TI agoraphobi* |
| S6 | AB phobi* |
| S5 | TI phobi* |
| S4 | AB panic |
| S3 | TI panic |
| S2 | TI obsessi* AB obsessi* or TI compulsi* or AB compulsi* |
| S1 | MH “Depression+” OR MH “Anxiety Disorders” OR MH “Anxiety” OR MH “Obsessive-Compulsive Disorder” OR MH “Panic Disorder” OR MH “Phobic Disorders” OR MH “Agoraphobia” OR MH “Claustrophobia” OR MH “Social Anxiety Disorders” OR MH “Stress Disorders, Post-Traumatic” OR TI depression or AB depression or TI depressive or AB depressive or TI dysthymi* or AB dysthymi* OR TI anxiety or AB anxiety or TI anxious AB anxious |

**Search 4. CINAHL database – Additional keyword searches**

| **#** | **Query** |
| --- | --- |
| S29 | S28 NOT S25 |
| S28 | S26 OR S27 |
| S27 | "Integrated depression care" OR "Integrated anxiety disorders care" OR "Stepped mental health care" OR "Stepped mental health service" OR "Stepped depression care" OR "Coordinated depression care" OR "Coordinated anxiety care" |
| S26 | "Collaborative mental health care" OR "Collaborative depression care" OR "Collaborative mental health intervention" OR "Collaborative depression intervention" OR "Collaborative mental health service" OR "Collaborative depression service" OR "Shared mental health care" OR "Shared depression care" OR "Shared depression management" OR "Integrated mental health service" OR "Integrated depression service" OR "Integrated mental health care" |
| S25 | S12 AND S23 AND S24 |
| S24 | MH “Quantitative Studies” OR MH ”Clinical Trials+“ OR MH ”Random Assignment“ OR PT ”Cinical Trial“ OR ( TI trial* or AB trial* ) OR ( TI ((singl* or doubl* or trebl* or tripl*) and (blind* or dummy or mask)) ) OR ( AB ((singl* or doubl* or trebl* or tripl*) and (blind* or dummy or mask)) ) OR ( TI (randomi?ed) or AB (randomi?ed) ) OR ( TI (random* and (allocat* or assign*)) or AB (random* and (allocat* or assign*)) ) OR ( TI (control* trial* or control* stud* or control group*) or AB (control* trial* or control* stud* or control group*) ) |
| S23 | S13 OR S14 OR S15 OR S16 OR S17 OR S18 OR S19 OR S20 OR S21 OR S22 |
| S22 | ( TI (care manage* or chronic care* or complex intervention* or cooperative behav* or co-operative behav* or joint working or pathway or interprofessional or inter-professional or interdisciplinary or inter-disciplinary or multidisciplin* or multi-disciplin* or multiprofession* or multi-profession* or transdisciplin* or trans-disciplin* or multifacet* or multi-facet* or complex intervention* or multiple intervention* or multi-intervention* or organisational intervention* or organizational intervention* or interpersonal relation” or inter-personal relation* or interinstitutional relation* or inter-institutional relation* or consultation liaison or algorithm* or treatment guideline* or treatment protocol* or treatment delivery or treatment model or adherence or compliance or concordance or patient care team or patient care management or patient care planning or case management or managed care program* or (healthcare N3 delivery) or (continuity N3 care) or professional-patient relations or interprofessional relations or inter-professional relations) ) OR ( AB (care manage* or chronic care* or complex intervention* or cooperative behav* or co-operative behav* or joint working or pathway or interprofessional or inter-professional or interdisciplinary or inter-disciplinary or multidisciplin* or multi-disciplin* or multiprofession* or multi-profession* or transdisciplin* or trans-disciplin* or multifacet* or multi-facet* or complex intervention* or multiple intervention* or multi-intervention* or organisational intervention* or organizational intervention* or interpersonal relation” or inter-personal relation* or interinstitutional relation* or inter-institutional relation* or consultation liaison or algorithm* or treatment guideline* or treatment protocol* or treatment delivery or treatment model or adherence or compliance or concordance or patient care team or patient care management or patient care planning or case management or managed care program* or (healthcare N3 delivery) or (continuity N3 care) or professional-patient relations or interprofessional relations or inter-professional relations) ) |
| S21 | TI "enhance* care*" or AB "enhance* care*" or TI "enhance* health*" or AB "enhance* health*" or TI "enhance* communicat*" or AB "enhance* communicat*" |
| S20 | TI "augment* care*" or AB "augment* care*" or TI "augment* health*" or AB "augment* health*" or TI "augment* communicat*" or AB "augment* communicat*" |
| S19 | TI "systematic care" or AB "systematic care" or TI "systematic health*" or AB "systematic health*" or TI "systematic work*" or AB "systematic work*" or TI "systematic interven*" or AB "systematic interven*" or TI "systematic service*" or AB "systematic service*" or TI "systematic model*" or AB "systematic model*" or TI "systematic effort*" or AB "systematic effort*" or TI "systematic manag*" or AB "systematic manag*" |
| S18 | TI "stepped care" or AB "stepped care" or TI "stepped health*" or AB "stepped health*" or TI "stepped work*" or AB "stepped work*" or TI "stepped interven*" or AB "stepped interven*" or TI "stepped service*" or AB "stepped service*" or TI "stepped model*" or AB "stepped model*" or TI "stepped effort*" or AB "stepped effort*" or TI "stepped manag*" or AB "stepped manag*" |
| S17 | TI "integrat* care" or AB "integrat* care" or TI "integrat* health*" or AB "integrat* health*" or TI "integrat* work*" or AB "integrat* work*" or TI "integrat* interven*" or AB "integrat* interven*" or TI "integrat* service*" or AB "integrat* service*" or TI "integrat* model*" or AB "integrat* model*" or TI "inte-grat* effort*" or AB "inte-grat* effort*" or TI "integrat* manag*" or AB "integrat* manag*" |
| S16 | TI "shared care" or AB "shared care" or TI "shared health*" or AB "shared health*" or TI "shared work*" or AB "shared work*" or TI "shared interven*" or AB "shared interven*" or TI "shared service*" or AB "shared service*" or TI "shared model*" or AB "shared model*" or TI "shared effort*" or AB "shared effort*" or TI "shared manag*" or AB "shared manag*" |
| S15 | TI "coordinat* care" or AB "coordinat* care" or TI "coordinat* health*" or AB "coordinat* health*" or TI "coordinat* work*" or AB "coordinat* work*" or TI "coordinat* interven*" or AB "coordinat* interven*" or TI "coordinat* service*" or AB "coordinat* service*" or TI "coordinat* model*" or AB "coordinat* model*" or TI "coordinat* effort*" or AB "coordinat* effort*" or TI "coordinat* manag*" or AB "coordinat* manag*" |
| S14 | TI "collaborat* care" AB "collaborat* care" or TI "collaborat* health*" or AB "collaborat* health*" or TI "collaborat* work*" or AB "collaborat* work* or TI "collaborat* interven*" or AB "collaborat* interven*" or TI "collaborat* service*" or AB "collaborat* service*" or TI "collaborat* model*" or AB "collaborat* model*" or TI "collaborat* effort*" or AB "collaborat* effort*" or TI "collaborat* manag*" or AB "collaborat* manag*" |
| S13 | MH “Case Mangement” OR MH Collaboration OR MH “Cooperative Behavior” OR MH “Interinstitutional Relations” OR MH “Interprofessional Relations” OR MH “Multidisciplinary Care Team” OR MH “Nurse-Physician Relations” OR MH ”Patient Compliance” OR MH “Patient Centered Care” OR MH “Pharmacists/UT” OR MH Teamwork |
| S12 | S1 OR S2 OR S3 OR S4 OR S5 OR S6 OR S7 OR S8 OR S9 OR S10 OR S11 |
| S11 | TI PTSD or AB PTSD or TI post-trauma* or AB post-trauma* or TI post trauma* or AB post-trauma* or TI postrauma* or AB postrauma* or TI posttrauma* or AB posttrauma* |
| S10 | TI GAD OR AB GAD |
| S9 | TI "social anxiety" or AB "social anxiety" |
| S8 | AB claustrophobi* OR TI claustrophobi* |
| S7 | AB agoraphobi* OR TI agoraphobi* |
| S6 | AB phobi* |
| S5 | TI phobi* |
| S4 | AB panic |
| S3 | TI panic |
| S2 | TI obsessi* AB obsessi* or TI compulsi* or AB compulsi* |
| S1 | MH “Depression+” OR MH “Anxiety Disorders” OR MH “Anxiety” OR MH “Obsessive-Compulsive Disorder” OR MH “Panic Disorder” OR MH “Phobic Disorders” OR MH “Agoraphobia” OR MH “Claustrophobia” OR MH “Social Anxiety Disorders” OR MH “Stress Disorders, Post-Traumatic” OR TI depression or AB depression or TI depressive or AB depressive or TI dysthymi* or AB dysthymi* OR TI anxiety or AB anxiety or TI anxious AB anxious |

**Search 5. WHO International Clinical Trials Registry Platform**

"collaborative care" OR "collaborative mental health care" OR "collaborative depression care" OR "collaborative model" OR "shared care" OR "shared mental health care"

"integrated care" OR "integrated mental health care" OR "integrated depression care" OR "integrated mental health service" OR "stepped care" OR "stepped depression care"

"stepped model" OR "case management" OR "care manager" OR "consultation-liaison"

**Search 6. ClinicalTrials.gov Registry**

"collaborative care" OR "collaborative mental health care" OR "collaborative depression care" OR "collaborative model" OR "shared care" OR "shared mental health care"

"integrated care" OR "integrated mental health care" OR "integrated depression care" OR "integrated mental health service" OR "stepped care" OR "stepped depression care"

"stepped model" OR "case management" OR "care manager" OR "consultation-liaison"

**Search 7. EU Clinical Trials Register**

"collaborative care" OR "collaborative mental health care" OR "collaborative depression care" OR "collaborative model" OR "shared care" OR "shared mental health care"

"integrated care" OR "integrated mental health care" OR "integrated depression care" OR "integrated mental health service" OR "stepped care" OR "stepped depression care"

"stepped model" OR "case management" OR "care manager" OR "consultation-liaison"
