# Supplementary material for: Personalized care planning and shared decision making in collaborative care programs for depression and anxiety disorders: A systematic review
Source: PLoS One. 2022 Jun 10;17(6):e0268649. doi: 10.1371/journal.pone.0268649 (PMC9187074; doi:10.1371/journal.pone.0268649)
Supplement: S2 File — (DOCX) [file pone.0268649.s002.docx]

**Supplementary materials**

**S2 File. Examples of personalized care planning and shared decision making processes**

| **Strategies and processes** | **Text extract** |
| --- | --- |
| **Personalized care planning** | |
| Preparation | “The first session will be 45 to 60 minutes long, during which the case manager will complete a detailed biopsychosocial patient assessment; elicit the patient’s ABC-E (autonomic, behavior, cognition, environment)) model about diabetes and/or CHD [*coronary heart disease*] and links with low mood; briefly educate the patient about diabetes/CHD and depression; assess risk; and introduce the treatment manual and develop with the patient a main problem statement.” (COINCIDE program)  “During the initial visit, the DCS [*depression care specialist*] conducted a clinical and psychosocial history, reviewed the educational materials, and discussed patient preferences for depression treatment (antidepressant medications or psychotherapy).” (IMPACT program) |
| Goal setting | “To establish a basis for collaborative problem definition and shared patient-provider treatment planning, the trauma support specialists were instructed to elicit and track patients’ posttraumatic concerns [13]. Thus on the surgical ward and at follow-up interviews patients were asked, “Of all the things that have happened to you since you were injured, what concerns you the most?” Patients’ posttraumatic concerns were incorporated into joint problem definition and the trauma support specialists were instructed to intervene on behalf of the patients in the resolution of these ﻿concerns whenever possible.” (Zatzick 2001 program)  “The HP [health professional] conducts the Cue and Response (C&R) interview with the veteran using open-ended questions to explore the same 14 items as the PIH [*Partners in Health questionnaire*]. This process leads to the veteran and HP deciding which of the 14 issues require intervention. The HP and veteran then identify the veteran’s main (life) Problem and set a medium-term (6–12 months) Goal (P&G). The output of these assessments is a set of issues documented on the care plan, which the veteran and HP agree will require their combined action over the following 12 months.” (FLINDERS program) |
| Action planning | “The ‘care manager’ works with both the patient and primary care physician and helps with developing a shared definition of the problem, providing patient education and support, developing a shared focus on specific problems, targeting goals and a specific action plan, offering support and problem-solving to optimize self-management, achieving closer monitoring of adherence and outcomes, and facilitating appointments to the primary care physician or specialist for patients with adverse outcomes or side-effects.” (Pathways program)  “The PCP [*primary care physician*] assessed depression in the context of other health care needs and initiated treatment planning, which included regular meetings with the care manager, prescribing medication, potential for outside psychotherapy referral, and follow-up.” (Weinreb program) |
| Documenting | “The remaining 15 minutes is set aside to add these data to the GP management plan (GPMP) before this is forwarded to the GP. The draft GPMP document becomes a readily accessible information support document for medication changes and referrals. The GP completes the plan during the consultation with the patient, providing the patient with a copy of the completed GPMP document.” (TrueBlue program)  “After GPs had commissioned patients to the trial, the nurses performed an initial assessment, checking patients’ perspectives, personal resources and their motivation for change. In cooperation with the patients, they developed specific objectives to be achieved over the course of the trial. Together, they decided on a hierarchy of goals, from smaller to larger ones, consented and recorded in written form.” (SMADS program) |
| Coordinating | “CMs [*care managers*] will coordinate care by facilitating the flow of key clinical information and recommendations between a centrally located mental health specialist (e.g., psychiatrist, psychologist – note that this specialist is collocated with telephone CMs) with expertise in PTSD and depression management, local clinic-based mental health specialists, the patient’s primary care provider and the patient.” (STEPS-UP program)  “Once treatment recommendations were created, the care manager worked to coordinate these recommendations with inpatient and outpatient medical care providers. The patient’s medical providers prescribed all antidepressant medication, and therapy referrals were coordinated through the medical teams.” (SUCCEED program) |
| Supporting | “The DCS [*depression care specialist*] nurse conducted the initial assessment, provided education and treatment, including support of antidepressant management and PST-PC [*problem solving therapy for primary care*], and provided proactive follow-up through visits and phone calls, tracking depression symptoms and treatment response through all phases of treatment. She consulted with the primary care providers, the team psychiatrist and other providers to adjust treatment plans as needed and to facilitate referrals.” (Pathways program)  “The main goals of the follow-up contacts were to systematically review progress and provide ongoing ﻿feedback and support. Patients were encouraged to adhere to their Depression Recurrence Prevention Plan in ‘good and in bad times’, and the prevention specialists boosted motivation and confidence in self-management skills by giving positive feedback, using motivational interviewing.” (Smit program) |
| Relapse prevention planning | “At the last face-to- face session, depression specialist and patient prepare a patient-tailored depression prevention plan, with the following topics: regular self-registration ﻿of early warning signs; stress reduction strategies; an ‘emergency plan’; and a medication plan, for patients using ADs [*antidepressants*]. A copy of this plan is sent to the PCP [*primary care physician*].” (Smit program)  “Once patients have reached remission, the CM [*care manager*] and patient work together towards a relapse prevention plan to help the patient identify when and where to seek help with future depressive symptoms or renewed problems for disease control.” (Teamcare PCN program) |
| Reviewing | “Intervention patients were seen for a baseline assessment, after one month and then two months later - i.e. three times over the initial 3 months of the study. The purpose of having the initial appointments more closely spaced was to allow sufficient time for the nurses to get to know the patient well and to formulate a clear management plan together. After this, the reviews for intervention patients took place 3 monthly for the remainder of the 24 month trial period, but could be more frequent if there were any significant clinical concerns about the patients’ mood.” (PROCEED program)  “The treatment plan included both pharmacological management of depression and psychosocial support. Following the VA and AHRQ treatment guidelines, particular attention was placed on assessments at 6 weeks, with recommendations to reevaluate the treatment plan for those who are unchanged or worse, to intensify or enhance treatment for those with partial responses (defined as more than a 30% decrease in depressive symptoms without meeting remission criteria [HDRS score ⬍10]), and to continue treatment for those with significant improvements. […] In addition to the training and the manuals, the BHSs [*behavioral health specialist*] met weekly with a psychiatrist to review each treatment plan. (Oslin program) |
| **Shared decision making** | |
| Identifying a decision | “Case managers should develop a collaborative relationship with patients. Patients are in charge of their own decisions. The case manager should always ensure these are made in an informed way. Case managers collaborate in these decisions by helping patients weigh up their options. Decisions will be about both medication issues and about behavioural activation activities.” (CADET program)  ﻿“The STEPS UP package is designed to give a menu of effective treatments and strategies available for patients with different levels of illness severity. Based on the patient’s initial assessment, the health care team in collaboration with the patient will identify the appropriate care step (e.g., STEP-1, STEP-2, STEP-3).” (STEPS UP program) |
| Exchanging information | ﻿“The nurses answered participants’ questions about current or past treatments and checked their concordance with the treatments they were currently receiving, clarifying any reasons for poor concordance. ﻿If there were current symptoms of depression, alternative or additional treatments were discussed. These could be pharmacological, psychological or social, with the rationale and evidence for any of these being made clear, both in the background literature given to patients and in their discussion with the nurses.” (PROCEED program)  “All patients receive an Anxiety Disorder Association of America brochure about their particular psychiatric condition prior to the care manager’s initial telephone call. The brochure aims to destigmatize the condition and briefly describe treatment options (guided “bibliotherapy,” pharmacotherapy, referral to a MHS, or some combination). The care manager reviews it with intervention patients during their initial telephone contact and provides an opportunity for them to ask questions. After collecting a detailed mental health history, including potentially contributing stressors (e.g., family, employment, and financial), use of any alcohol, caffeine, over-the-counter medications (e.g., pseudoephedrine), or herbal supplements being used to self-medicate anxiety symptoms, the care manger elicits each patient’s treatment preference(s)…” (Rollman 2005 program) |
| Presenting options | “Therefore, in keeping with principles of shared decision-making, we provided patients with oral and written educational materials about their condition (‘bibliotherapy’), and offered a variety of treatment options: (1) initiation or adjustment of antidepressant pharmacotherapy provided under their PCP’s [*primary care provider’s*] direction; (2) referral to a community MHS [*mental health service*]; (3) a combination of the above; or (4) “watchful-waiting” if the patient’s mood symptoms were only mildly elevated (PHQ-9 score of 10–14) and he/she had no prior history of depression (Table 2).” (Bypassing the Blues program)  “Primary care clinicians seldom have the time, motivation, and knowledge to explain the range of PTSD and depression treatment options to patients. CMs [*case managers*] will therefore assist the primary care clinician by providing information related to treatment options and selecting the “best step” or the “best next step” (if the current treatment is not working) for a patient’s treatment plan. CMs will provide patient education regarding treatment options and will troubleshoot patient concerns, questions, side-effects, or problems related to specific treatment options. […] Examples of treatment options include combined case management, psychotherapy, and pharmacotherapy.” (STEPS UP program) |
| Exploring preferences | “At this 1 week visit, the nurse assessed the nine criteria for major depression, evaluated the patient’s treatment preferences (pharmacotherapy, psychotherapy, watchful waiting), and addressed identified barriers to care by negotiating that patients complete a small assignment to ﻿increase or maintain their readiness to engage in active treatment.” (Rost QID program)  ﻿“Women randomized to the intervention had an initial engagement session with a depression care manager designed to provide education about depression, elicit health concerns and barriers to treatment, and enhance participation in depression treatment. During the subsequent session, depression care managers obtained clinical history, reviewed educational materials, and described and discussed patient preferences for initiating treatment with either antidepressant medication or problem-solving treatment–primary care.” (DAWN program) |
| Discussing pros and cons | “For women requesting antidepressants as an initial treatment, the DCS [*depression care specialist*] encouraged them to engage in a risk-benefit decision-making process [29] with their OB [*obstetrician*] provider to discuss the risks of both antidepressants [30] and untreated depression during pregnancy and lactation.[31, 32] Once a woman made an informed decision, the study psychiatrist via the DCS made recommendations to her OB provider (usually for a selective serotonin reuptake inhibitor), based on a clinical algorithm, incorporating the patient’s current medication and/or past response to antidepressants.” (MOMcare program)  “When patients have been prescribed medication by their GPs, case managers should ascertain how closely the patient wishes to follow the GP‛s prescription. Where a patient does not wish to adhere to the GP‛s prescription, the case manager should respect the patient‛s view even if they disagree, and help the patient to weigh up the pros and cons of their decision. Case managers must ensure patients‛ decisions are informed by accurate educational input on antidepressant action and respect and support decisions.” (CADET program) |
| Making a decision together | ﻿“CBT began with an initial ‘choice’ session, which reviewed the basic therapy approach and identified the adolescent’s issues and goals. The youth and therapist jointly chose one of two therapy approaches to try first: either four sessions of cognitive restructuring or four sessions of behavioral activation.” (Clarke program)  ﻿“Unlike the structured and planned visits and activities as specified in the care management group, the physician and the patient in the enhanced physician care group (25 participants) jointly decided which treatment regimen they considered appropriate, and how often and when the patient would receive follow-up.” (Kwong program) |
| Revisiting a decision | “Nurses interacted regularly (via written notes and verbally) with the primary care physician treating the patient. On alternate weeks, nurses reviewed cases by telephone with the psychiatrist supervisor. The psychiatrist supervisor regularly reviewed choices and dosages of medication and clinical response and recommended changes, which the nurse discussed with the primary care physician and patient.” (Pathways program)  “Later, if appropriate, discussions about treatment decisions can be initiated by case managers, for example where symptoms and/or PHQ-9 scores do not improve.” (CADET program) |

**References**

À ajouter
